# Supplementary material for: Cardiovascular health metrics from mid- to late-life and risk of dementia: A population-based cohort study in Finland
Source: PLoS Med. 2020 Dec 15;17(12):e1003474. doi: 10.1371/journal.pmed.1003474 (PMC7737898; doi:10.1371/journal.pmed.1003474)
Supplement: S1 Study protocol — (DOCX) [file pmed.1003474.s002.docx]

**S1 Study Protocol**

The present study is part of the ongoing Cardiovascular Risk Factors, Aging and Dementia (CAIDE) study in Kuopio, Finland [1,2]. The CAIDE study aims to investigate the association of social, lifestyle, and cardiovascular risk factors with cognitive function, dementia, and structural changes in the brain. More details of the study design can be found at [www.uef.fi/caide/](http://www.uef.fi/caide/). The study protocol of CAIDE was approved by the local ethics committee at Kuopio University and the Kuopio University Hospital in Kuopio, Finland as well as the ethics committee at Karolinska Institutet in Stockholm, Sweden. The verbal informed consent (midlife surveys in 1972-1987) or the written informed consent (late-life surveys in 1998 and 2005-2008) was obtained from all participants prior to each wave of the examination.

The present study was planned within the framework of the CAIDE study aiming to determine the associations of cardiovascular health (CVH) from midlife to late-life with the risk of dementia. In May 2019, we initiated this analysis. Our hypothesis was that optimal CVH metrics, especially occurring from midlife onwards, were associated with a reduced risk of incident dementia developed later in life. The research questions and the plan of data analysis have been approved by the PI and research team of the CAIDE study.

**Research questions:**

1. Are the composite CVH metrics measured in midlife and late-life differentially associated with the risk of dementia?
2. To what extent are the changes or patterns of CVH metrics from midlife to late-life associated with the risk of dementia in late-life?

**Planned statistical analyses**

1. Performing the comparison of baseline characteristics in midlife between participants included in the analytical sample and those lost to follow-up.
2. Calculating the incidence of dementia (number of incident dementia cases divided by the total person-years of follow-ups) by levels of the composite global CVH metrics.
3. Performing Cox regression analysis to assess the associations of global CVH metrics in midlife and late-life as well as their patterns from midlife to late-life with the risk of dementia, while controlling for potential confounding factors.
4. Estimating the population attributable risk of global CVH metrics for dementia.

**Performed analyses**

1. We compared both midlife (1972-1987) and late-life (1998) characteristics between participants included in the analytical sample and those lost to follow-up.
2. We calculated the incidence rates of dementia using person-years of follow-ups and performed the Cox proportional-hazards models to estimate the cumulative incidence of dementia by taking into account death as a competing risk event.
3. We performed Cox regression analysis to assess the associations of the composite global, behavioral, and biological CVH metrics in midlife and late-life as well as the patterns of CVH metrics from midlife to late-life with the risk of dementia in late-life.
4. We performed the Cox regression analysis to assess the associations of individual components of CVH metrics in midlife and late-life with risk of dementia in late-life.
5. We performed the Fine and Gray competing risk regression analysis to assess the associations of the composite CVH metrics in midlife, late-life, and the patterns from midlife to late-life with the risk of dementia after controlling for multiple potential confounders as well as taking into account the competing risk due to death.

**Differences in analyses between planned and performed**

1. In response to the reviewer’s comments and suggestions, we performed Cox proportional-hazards models to estimate the cumulative incidence of dementia to take into account the competing risk due to death.
2. In response to the reviewer’s comments and suggestions, we examined the associations of individual CVH metric components as well as composite global, behavioral, and biological CVH metrics in midlife and late-life with dementia risk in late-life.
3. In response to the reviewer’s comments, we performed analysis to assess the possible statistical interaction between midlife and late-life CVH metrics on the risk of dementia. Then, we further assessed the patterns of composite global, behavioural, and biological CVH metrics in midlife and late-life in association with the risk of dementia.
4. In response to the reviewer’s comments and suggestions, Fine and Gray competing risk regression analysis was performed to examine the associations between midlife or late-life CVH metrics and risk of dementia while taking into account the competing risk of death.
5. Due to the unexpected complex relationships of individual and composite CVH metrics in midlife and late-life with dementia risk, we did not estimate the population attributable risk of global CVH metrics for dementia.

**References**

1. Kivipelto M, Helkala EL, Laakso MP, Hänninen T, Hallikainen M, Alhainen K, et al. Midlife vascular risk factors and Alzheimer's disease in later life: longitudinal, population based study. BMJ. 2001;322:1447-51.

2. Barbera M, Kulmala J, Lisko I, Pietilä E, Rosenberg A, Hallikainen I, et al. Third follow-up of the Cardiovascular Risk Factors, Aging and Dementia (CAIDE) cohort investigating determinants of cognitive, physical, and psychosocial wellbeing among the oldest old: the CAIDE85+ study protocol. BMC Geriatr. 2020;20(1):238.
