# Supplementary material for: Cardiovascular health metrics from mid- to late-life and risk of dementia: A population-based cohort study in Finland
Source: PLoS Med. 2020 Dec 15;17(12):e1003474. doi: 10.1371/journal.pmed.1003474 (PMC7737898; doi:10.1371/journal.pmed.1003474)
Supplement: S1 Statistical methods — (DOCX) [file pmed.1003474.s003.docx]

**S1 Statistical methods. Fine and Gray competing risk regression model**

Fine and Gray competing risk regression model has been often used to estimate the cause-specific hazard in the competing-risk setting [1]. In this study, we used the Fine and Gray competing risk model to take into account death as a competing event when we assessed the associations of global, behavioral, and biological CVH metrics with dementia. The hypothesis was based on the proportional subdistribution hazard, which is a measure of association that is due to both the association of the exposure (e.g., CVH metrics) with the event of interest (e.g., dementia) and the possibly differential impact of competing events (e.g., death) on the risk set for individuals with different level of the exposure (e.g., CVH metrics).

The risk sets at different lengths of follow-up were constructed by including individuals without any event as well as those with the competing event (e.g., death) [2]. For example, death was not considered as censored, and one individual died at a time point and was still maintained in the subsequent risk sets. Therefore, with the increase of follow-up time, the risk sets comprised an increasing proportion of participants who have developed the competing event (e.g., death). With this structure, a subdistribution hazard f unction (i.e., cumulative incidence function) is defined as the probability of an event of interest given that the individuals have survived up to a time point without any event or have had the competing event prior to that time point [2].

Based on the theory and methods described above, we used the following SAS program to perform the Fine and Gray compering risk regression analysis:

**proc** **phreg** data=<database>;

class var <exposure covariates> / order=internal ref=first param=glm;

model time*outcome(**0**)= var <exposure covariates> /eventcode=**1** <0=censor, 1=outcome event, 2=competing event>;

hazardratio 'cause-specific hazards' var <exposure> / diff=all;

**run**;

**References**

1. Fine JP, Gray RJ. A proportional hazards model for the subdistribution of a competing risk. J Am Stat Assoc. 1999;94(446):496-509.

2. Lau B, Cole SR, Gange SJ. Competing risk regression models for epidemiologic data. Am J Epidemiol. 2009;170(2):244-56.
