# Supplementary material for: Cardiovascular health metrics from mid- to late-life and risk of dementia: A population-based cohort study in Finland
Source: PLoS Med. 2020 Dec 15;17(12):e1003474. doi: 10.1371/journal.pmed.1003474 (PMC7737898; doi:10.1371/journal.pmed.1003474)
Supplement: S1 Table — *The number of participants with missing data was 13 persons for physical activity, and these individuals were included in the analysis by creating a dummy variable to indicate those with missing values. #In midlife, information on fasting plasma glucose was lacking, instead, the information on self-reported history of diabetes, a recorded diagnosis of diabetes from the inpatient register, and the use of antidiabetic medication from the prescribed drug register were used as a proxy. §Model 1 was adjusted for age, sex, education, and other components in the table; model 2 was additionally adjusted for APOE ε4 allele and cardiovascular disease in midlife; and model 3 included death as a competing risk event with the adjustment of all covariates in model 2. APOE, apolipoprotein E; CI, confidence interval; CVH, cardiovascular health; HR, hazard ratio. (DOCX) [file pmed.1003474.s004.docx]

**S1 table. The association of individual cardiovascular health metrics in midlife (1972-1987) with risk of dementia detected in late-life (both 1998 and 2005-2008) (n=1449)**

| **Midlife cardiovascular health metrics^*^** | **No. of subjects** | **No. of dementia cases** | **Model 1^§^** | | **Model 2^§^** | | **Model 3^§^** | |
| --- | --- | --- | --- | --- | --- | --- | --- | --- |
|  |  |  | **HR (95% CI)** | **p** | **HR (95% CI)** | **p** | **HR (95% CI)** | **p** |
| Smoking |  |  |  |  |  |  |  |  |
| Poor | 61 | 7 | 1.00 (reference) |  | 1.00 (reference) |  | 1.00 (reference) |  |
| Intermediate | 290 | 23 | 0.03 (0.01, 0.07) | <0.001 | 0.03 (0.01, 0.07) | <0.001 | 0.41 (0.14, 1.14) | 0.088 |
| Ideal | 1098 | 78 | 0.06 (0.02, 0.14) | <0.001 | 0.06 (0.02, 0.13) | <0.001 | 0.40 (0.15, 1.02) | 0.056 |
| Physical activity |  |  |  |  |  |  |  |  |
| Poor | 51 | 5 | 1.00 (reference) |  | 1.00 (reference) |  | 1.00 (reference) |  |
| Intermediate | 772 | 66 | 0.73 (0.29, 1.86) | 0.510 | 0.79 (0.30, 2.11) | 0.636 | 1.33 (0.50, 3.55) | 0.571 |
| Ideal | 597 | 35 | 0.71 (0.27, 1.86) | 0.484 | 0.75 (0.27, 2.06) | 0.579 | 0.96 (0.35, 2.67) | 0.942 |
| Body mass index |  |  |  |  |  |  |  |  |
| Poor | 241 | 31 | 1.00 (reference) |  | 1.00 (reference) |  | 1.00 (reference) |  |
| Intermediate | 718 | 49 | 0.75 (0.46, 1.22) | 0.247 | 0.76 (0.46, 1.24) | 0.267 | 0.69 (0.42, 1.14) | 0.143 |
| Ideal | 490 | 28 | 0.48 (0.27, 0.86) | 0.014 | 0.49 (0.27, 0.87) | 0.015 | 0.66 (0.38, 1.13) | 0.132 |
| Plasma glucose^#^ |  |  |  |  |  |  |  |  |
| Poor | 55 | 6 | 1.00 (reference) |  | 1.00 (reference) |  | 1.00 (reference) |  |
| Intermediate | 3 | 0 | - | - | - | - | - | - |
| Ideal | 1391 | 102 | 0.39 (0.16, 0.94) | 0.036 | 0.43 (0.18, 1.05) | 0.064 | 0.56 (0.21, 1.51) | 0.253 |
| Total cholesterol |  |  |  |  |  |  |  |  |
| Poor | 944 | 81 | 1.00 (reference) |  | 1.00 (reference) |  | 1.00 (reference) |  |
| Intermediate | 384 | 19 | 0.75 (0.44, 1.27) | 0.281 | 0.76 (0.45, 1.30) | 0.318 | 0.76 (0.45, 1.30) | 0.351 |
| Ideal | 121 | 8 | 0.90 (0.41, 1.98) | 0.788 | 0.90 (0.41, 1.98) | 0.794 | 1.25 (0.60, 2.61) | 0.549 |
| Blood pressure |  |  |  |  |  |  |  |  |
| Poor | 618 | 59 | 1.00 (reference) |  | 1.00 (reference) |  | 1.00 (reference) |  |
| Intermediate | 760 | 45 | 0.91 (0.60, 1.39) | 0.662 | 0.92 (0.60, 1.41) | 0.700 | 0.94 (0.62, 1.42) | 0.758 |
| Ideal | 71 | 4 | 1.20 (0.42, 3.40) | 0.730 | 1.22 (0.43, 3.44) | 0.714 | 0.89 (0.31, 2.53) | 0.828 |

^*^The number of subjects with missing data was 13 for physical activity, and these individuals were included in the analysis by creating a dummy variable to indicate those with missing values.

^#^In midlife, information on fasting plasma glucose was lacking, instead, the information on self-reported history of diabetes, a recorded diagnosis of diabetes from the inpatient register, the use of antidiabetic medication from the prescribed drug register was used as a proxy.

^§^Model 1 was adjusted for age, sex, education, and other components in the table; model 2 was additionally adjusted for APOE ε4 allele and cardiovascular disease in midlife; model 3 included death as a competing risk event with the adjustment of all covariates in model 2.

Abbreviations: HR, hazard ratio; CI, confidence interval.
