# Supplementary material for: Cardiovascular health metrics from mid- to late-life and risk of dementia: A population-based cohort study in Finland
Source: PLoS Med. 2020 Dec 15;17(12):e1003474. doi: 10.1371/journal.pmed.1003474 (PMC7737898; doi:10.1371/journal.pmed.1003474)
Supplement: S2 Table — *The number of participants with missing data was 6 persons for smoking and 1 person for physical activity, and these individuals were included in the analysis by creating a dummy variable for each factor to indicate those with missing values. #Model 1 was adjusted for age, sex, education, and other components in the table; model 2 was additionally adjusted for APOE ε4 allele and cardiovascular disease in late life; and model 3 included death as a competing risk event with the adjustment of all covariates in model 2. APOE, apolipoprotein E; CI, confidence interval; CVH, cardiovascular health; HR, hazard ratio. (DOCX) [file pmed.1003474.s005.docx]

**S2 table. The association of individual cardiovascular health metrics in late-life (1998) with risk of dementia detected in late-life (2005-2008) (n=744)**

| **Late-life cardiovascular health metrics^*^** | **No. of subjects** | **No. of dementia cases** | **Model 1^#^** | | **Model 2^#^** | | **Model 3^#^** | |
| --- | --- | --- | --- | --- | --- | --- | --- | --- |
|  |  |  | **HR (95% CI)** | **p** | **HR (95% CI)** | **p** | **HR (95% CI)** | **p** |
| Smoking |  |  |  |  |  |  |  |  |
| Poor | 34 | 2 | 1.00 (reference) |  | 1.00 (reference) |  | 1.00 (reference) |  |
| Intermediate | 198 | 13 | 0.75 (0.16, 3.46) | 0.709 | 0.72 (0.15, 3.35) | 0.671 | 0.99 (0.14, 7.20) | 0.990 |
| Ideal | 506 | 31 | 0.71 (0.16, 3.09) | 0.650 | 0.70 (0.16, 3.05) | 0.634 | 0.76 (0.12, 5.00) | 0.779 |
| Physical activity |  |  |  |  |  |  |  |  |
| Poor | 27 | 7 | 1.00 (reference) |  | 1.00 (reference) |  | 1.00 (reference) |  |
| Intermediate | 123 | 8 | 0.75 (0.24, 2.41) | 0.632 | 0.69 (0.21, 2.28) | 0.539 | 0.86 (0.24, 3.11) | 0.817 |
| Ideal | 593 | 32 | 0.49 (0.19, 1.28) | 0.144 | 0.51 (0.19, 1.36) | 0.178 | 0.50 (0.17, 1.42) | 0.192 |
| Body mass index |  |  |  |  |  |  |  |  |
| Poor | 192 | 11 | 1.00 (reference) |  | 1.00 (reference) |  | 1.00 (reference) |  |
| Intermediate | 378 | 22 | 1.32 (0.62, 2.83) | 0.472 | 1.44 (0.67, 3.12) | 0.349 | 1.14 (0.54, 2.41) | 0.730 |
| Ideal | 174 | 14 | 1.45 (0.63, 3.33) | 0.384 | 1.59 (0.68, 3.71) | 0.280 | 1.29 (0.44, 3.72) | 0.642 |
| Plasma glucose |  |  |  |  |  |  |  |  |
| Poor | 31 | 3 | 1.00 (reference) |  | 1.00 (reference) |  | 1.00 (reference) |  |
| Intermediate | 90 | 5 | 0.47 (0.11, 2.07) | 0.319 | 0.50 (0.11, 2.23) | 0.360 | 0.51 (0.11, 2.41) | 0.399 |
| Ideal | 623 | 39 | 0.46 (0.13, 1.55) | 0.208 | 0.47 (0.13, 1.63) | 0.233 | 0.61 (0.14, 2.69) | 0.512 |
| Total cholesterol |  |  |  |  |  |  |  |  |
| Poor | 265 | 14 | 1.00 (reference) |  | 1.00 (reference) |  | 1.00 (reference) |  |
| Intermediate | 369 | 21 | 1.27 (0.63, 2.57) | 0.500 | 1.37 (0.66, 2.84) | 0.391 | 1.45 (0.63, 3.34) | 0.384 |
| Ideal | 110 | 12 | 1.65 (0.71, 3.83) | 0.242 | 1.79 (0.76, 4.23) | 0.184 | 2.34 (0.80, 6.87) | 0.120 |
| Blood pressure |  |  |  |  |  |  |  |  |
| Poor | 276 | 13 | 1.00 (reference) |  | 1.00 (reference) |  | 1.00 (reference) |  |
| Intermediate | 451 | 33 | 1.74 (0.88, 3.42) | 0.109 | 1.86 (0.91, 3.81) | 0.088 | 1.97 (0.93, 4.17) | 0.078 |
| Ideal | 17 | 1 | 1.67 (0.21, 13.22) | 0.626 | 1.82 (0.23, 14.57) | 0.572 | 2.28 (0.24, 21.82) | 0.475 |

^*^The number of subjects with missing data was 6 for smoking and 1 for physical activity, and these individuals were included in the analysis by creating a dummy variable for each factor to indicate those with missing values.

^#^Model 1 was adjusted for age, sex, education, and other components in the table; model 2 was additionally adjusted for APOE ε4 allele and cardiovascular disease in late-life; model 3 included death as a competing risk event with the adjustment of all covariates in model 2.

Abbreviations: HR, hazard ratio; CI, confidence interval.
