# Supplementary material for: Cardiovascular health metrics from mid- to late-life and risk of dementia: A population-based cohort study in Finland
Source: PLoS Med. 2020 Dec 15;17(12):e1003474. doi: 10.1371/journal.pmed.1003474 (PMC7737898; doi:10.1371/journal.pmed.1003474)
Supplement: Attachment — Submitted filename: Response to reviews_2020Aug25.doc [file pmed.1003474.s006.doc]

**A brief summary of the major revisions:**

1. We updated our database by adding data from the patient register and the prescribed drug register, as a result, several additional subjects with diabetes in midlife have been identified. Accordingly, we have now completely updated all the results by re-running our previous analyses.
2. Inspired by the reviewers’ comments, we now further separated global cardiovascular health (CVH) metrics into behavioural CVH metrics and biological CVH metrics in all our analyses. As a result, extensive revisions have been made throughout the manuscript.
3. We performed complete analysis to examine the changes or patterns of global, behavioural, and biological CVH metric levels (poor, intermediate, and ideal levels) from middle age to late-life in association with late-life risk of dementia, and reported the full results in Fig 2.
4. In response to the reviewer’s comments and suggestions, we provided supplemental tables to show results of individual CVH metric components in midlife and late-life in relation to dementia risk (S1 and S2 Tables).

Editors’ comments

1. In the last sentence of the Abstract Methods and Findings section, please include a brief note about any key limitation(s) of the study's methodology.

Answer: We have now added one sentence in the Methods and Findings section to specify the key limitations of the study’s methodology (see page 4).

2. At this stage, we ask that you include a short, non-technical Author Summary of your research to make findings accessible to a wide audience that includes both scientists and non-scientists (I noted this had been provided as a supplementary file, please include the text immediately following the Abstract in your revised manuscript). This text is subject to editorial change and should be distinct from the scientific abstract.

Answer: Author Summary has been added following the Abstract (see page 5).

3. We'd suggest using the STROBE guideline to assist/enhance study reporting - if doing this please include the completed STROBE checklist as Supporting Information. Please add the following statement, or similar, to the Methods: "This study is reported as per the Strengthening the Reporting of Observational Studies in Epidemiology (STROBE) guideline (SChecklist)." When completing the checklist, please use section and paragraph numbers, rather than page numbers.

Answer: The statement of using the STROBE guidelines has been added to the Methods section, as suggested (see page 8). The STROBE checklist has been completed and added as Supporting Information (S1 Checklist).

4. In the Methods section of your paper, please clarify if the study had a prospective protocol or analysis plan? Please state this (either way) early in the Methods section.

a) If a prospective analysis plan (from your funding proposal, IRB or other ethics committee submission, study protocol, or other planning document written before analyzing the data) was used in designing the study, please include the relevant prospectively written document with your revised manuscript as a Supporting Information file to be published alongside your study, and cite it in the Methods section. A legend for this file should be included at the end of your manuscript.

b) If no such document exists, please make sure that the Methods section transparently describes when analyses were planned, and when/why any data-driven changes to analyses took place.

c) In either case, changes in the analysis-- including those made in response to peer review comments-- should be identified as such in the Methods section of the paper, with rationale.

Answer: We have now provided the prospective protocol (analysis plan) as a Supporting information (S1 Study Protocol). The statement has been added to the section of Statistical analysis (see page 11).

**Reviewer #1**

"Cardiovascular health metrics from mid- to late-life and risk of dementia: a population-based study" attempts to quantify the association of cardiovascular health metrics (CVH) with the risk of incident dementia, on 1449 participants from the Finnish Cardiovascular Risk Factors, Aging and Dementia (CAIDE) study. Six AHA metrics (excluding diet) were used to represent CVH, with the DSM-IV criteria used to diagnose dementia, with Cox regression models used for the analysis. It was concluded that either maintaining lifelong good CVH, or improving CVH from mid to late lift, is associated with substantially reduced risk of dementia.

The usage of AHA-based CVH metrics follows prior studies ([17]-[19]) with similar structures, while the DSM-IV criteria for diagnosing dementia is also fairly long-established. The study sample size of 1449 participants is comparable to the three works referenced above (1033-6626 participants), as is the regression analysis. A major contribution of this paper over prior works pertains to the examination of mid-to-late-life CVH metrics, over late-life CVH metrics alone, which has yielded mixed results in the previous works.

This manuscript thus represents a new datapoint in CVH to dementia association, that clearly builds upon a coherent body of previous research. However, while the total number of participants is in the ballpark of previous studies, the number of positive dementia diagnoses is relatively low (61 in 1998, 47 in 2005-08). This is further reflected in the especially low number of valid cases for some stratifications (particularly poor/poor & the novel midlife/late-life analysis, Table 4), and correspondingly wide hazard ratio confidence intervals.

Answer: We thank the reviewer for the overall positive comments as well as valuable suggestions to improve our work.

1. The definition of the two late-life examinations (1998, 2005-08) was not very easy to follow in the Study Design section of the main text, possibly due to the explanation being relegated to the secondary caption of the Figure 1 flowchart. This might be shifted to the main text.
Answer: We have incorporated the notes from Fig 1 into the text section of the Study design and participants (see page 8).

2. The likely relatively large number of participants who died before they could be included in the analytical samples, would appear to have possibly affected the statistics/conclusions. From the main text, it would appear that patients who manifested dementia, but died before a follow-up examination, would be ignored. While dementia incidence might be unknown for these cases, it might be appropriate to consider a survival model for dementia/death, or possibly include fraction/age of death at follow-up in the demographics (e.g. as done in "Midlife cardiovascular fitness and dementia: A 44-year longitudinal population study in women", Horder et al., Neurology, 2018)

Answer: We completely agree with the reviewer that some people (including those who developed dementia) in the middle aged cohort (age 45-64 years) might have died before they could survive to 1998 when the first late-life examination was performed. We were not able to evaluate the impact of the selective survival prior to the first late-life examination (1998) due to lack of relevant data (e.g., diagnosis of dementia), but given the relatively young age, it was expected that relatively few people might have developed late-onset dementia. However, we could partially evaluate the impact of selective survival from the first late-life examination onward by estimating the cumulative incidence of dementia from Cox models after taking into account death as a competing risk as well as by examining the associations of CVH metrics with dementia risk with the Fine and Gray competing risk models (death as a competing event). We have now briefly described this analysis in the methods section (see page 12) and reported the results from this analysis (see pages 13-15, model 3 in Tables 3-4, Fig 2, and S1-S2 tables) as well as briefly discussed the possible impact on the results and acknowledged it as a potential limitation (see page 19).

3. The adjustment for the APOE genotype in the context of CVH/dementia might be briefly motivated in terms of possible protective effect.

Answer: We have added a brief description in Statistical analysis about the rationale and motivation of controlling for APOE genotype in our analysis (see page 12).

4. The definition for "history of cardiovascular disease", as displayed in Table 1, might be specified in greater detail.
Answer: We have added more information about how we defined cardiovascular disease and what specific cardiovascular diseases we included in the section of Data collection and definitions (page 9).

5. The full implementation of intervention assumption for population attributable risk (PAR %) in Page 8 might be more fully elaborated. In particularly, what exactly are these interventions, and what is their expected success rate?
Answer: We have removed population attributable risk (PAR%) from the revised manuscript because: (1) cardiovascular health metrics (exposures) are composite measurements of multiple factors and there appears to have a complex relationship between the composite CVH metrics and risk of dementia; (2) the strength of association between CVH metric measures and risk of dementia might be underestimated and varied with age when the exposures are assessed; and (3) the possible interactions among individual CVH metric components as well as between midlife and late-life CVH metric measurements on the risk of dementia may further complicate the interpretations of PAR%.

6. For Table 4, "Intermediate or ideal (≥6) in either midlife or late-life" might be clarified as "either midlife or late-life only". Moreover, since improved CVH (i.e. poor in mid-life, intermediate or ideal in late-life) is discussed in the main text, it might also be shown in Table 4 (possibly along with intermediate/ideal in mid-life, poor in late-life)

Answer: We thank the reviewer for the extremely valuable comments, which inspired us to thoroughly explore the changes in global CVH metrics from mid- to late-life in association with risk of late-life dementia. Therefore, we have now replaced Table 4 with a figure (Fig 2) showing the hazard ratios (95% CI) of dementia associated with the different combinations of three levels of global CVH metrics (poor, intermediate and ideal levels) in mid- and late-life (Fig 2A). Similarly, we showed the results for behavioral and biological CVH metrics in the Fig 2B and 2C. The figure shows the full picture of the changes or patterns of composite CVH metrics from mid- to late-life in relation to dementia risk in late-life. Accordingly, we have now specified this analysis in the methods (see page 11), results (see pages 14-15), and discussion sections (see pages 17-18).

In addition, to further address the reviewer’s specific comments, we have provided the results in a table on the next page concerning the associations between the combinations of certain levels of global, behavioral, and biological CVH metrics in midlife and late-life in association with the risk of dementia. Because this table does not add more information than Fig 2, we decided to show the results only in this response letter, not in the manuscript (neither as a supplemental table).

7. While it is stated that "There was no association between CVH metrics and dementia risk when CVH metrics were assessed in late-life" (Page 10), Table 3 would seem to suggest an overall risk reduction, were the Intermediate & Ideal categories combined and compared against Poor; the authors might also comment on Intermediate & Ideal being analyzed separately in Tables 2 & 3, but combined for the analysis in Table 4.
Answer: We thank the reviewer for the comments. We analysed the data by combining intermediate and ideal categories (vs. poor), as the reviewer suggested. We found no significant association between late-life CVH metrics and dementia: 0.75 (0.29, 1.93) in model 1, 0.93 (0.33, 2.63) in model 2, and 0.78 (0.32-1.94) in model 3. In response to the reviewer’s comment no. 6 above, when we assessed the change or pattern of CVH metrics (global, behavioral, and biological CVH metrics) from midlife to late-life in association with dementia, we have now added Fig 2 to show the different combinations of CVH groups from midlife to late-life (Fig 2, also refer to our response to the comment no. 6 above).

**Table. The change of cardiovascular health metrics from midlife (1972-1987) to late life (1998) and the risk of dementia detected in late life (2005-2008) (n=7**44)

| **CVH metrics in midlife and late-life** | **No. of subjects** | **No. of dementia cases** | **Person-years of follow-up** | **Incidence rate (per 1000 person-years)** | **Cumulative incidence rate (%)^*^** | **Hazard ratio (95% confidence interval)** | | |
| --- | --- | --- | --- | --- | --- | --- | --- | --- |
|  |  |  |  |  |  | **Model 1^#^** | **Model 2^#^** | **Model 3^#^** |
| **Global CVH metrics** |  |  |  |  |  |  |  |  |
| Poor in both midlife & late-life | 20 | 4 | 549 | 7.28 | 15.18 | 1.00 (ref.) | 1.00 (ref.) | 1.00 (ref.) |
| Poor in midlife, intermediate or ideal in late-life | 55 | 5 | 1496 | 3.34 | 6.63 | 0.55 (0.14, 2.13) | 0.48 (0.12, 1.84) | 0.73 (0.18, 3.04) |
| Intermediate or ideal in midlife, poor in late-life | 46 | 2 | 1354 | 1.48 | 2.58 | 0.23 (0.04, 1.31) | 0.12 (0.02, 0.93) | 0.32 (0.05, 1.96) |
| Intermediate or ideal in both midlife and late-life | 623 | 36 | 18165 | 1.98 | 3.71 | 0.34 (0.12, 0.98) | 0.32 (0.11, 0.93) | 0.50 (0.14, 1.85) |
| **Behavioral CVH metrics** |  |  |  |  |  |  |  |  |
| Poor in both midlife & late-life | 13 | 3 | 357 | 8.40 | 21.18 | 1.00 (ref.) | 1.00 (ref.) | 1.00 (ref.) |
| Poor in midlife, intermediate or ideal in late-life | 27 | 4 | 706 | 5.66 | 16.14 | 0.52 (0.11, 2.41) | 0.51 (0.11, 2.42) | 0.79 (0.16, 3.78) |
| Intermediate or ideal in midlife, poor in late life | 23 | 2 | 682 | 2.93 | 6.25 | 0.19 (0.03, 1.18) | 0.20 (0.03, 1.25) | 0.21 (0.02, 2.78) |
| Intermediate or ideal in both midlife and late life | 681 | 38 | 19820 | 1.92 | 3.84 | 0.18 (0.05, 0.58) | 0.18 (0.06, 0.61) | 0.13 (0.03, 0.56) |
| **Biological CVH metrics** |  |  |  |  |  |  |  |  |
| Poor in both midlife & late-life | 66 | 3 | 1915 | 1.57 | 3.55 | 1.00 (ref.) | 1.00 (ref.) | 1.00 (ref.) |
| Poor in midlife, intermediate or ideal in late life | 135 | 10 | 3881 | 2.58 | 5.46 | 1.44 (0.40, 5.28) | 1.40 (0.38, 5.14) | 2.02 (0.58, 7.06) |
| Intermediate or ideal in midlife, poor in late life | 105 | 4 | 3119 | 1.28 | 2.63 | 0.89 (0.19, 4.09) | 0.69 (0.14, 3.39) | 1.41 (0.30, 6.65) |
| Intermediate or ideal in both midlife and late life | 438 | 30 | 12650 | 2.37 | 3.79 | 1.88 (0.56, 6.26) | 1.93 (0.58, 6.43) | 2.27 (0.60, 8.58) |

^*^The cumulative incidence was calculated from the Cox regression models after taking into account the competing risk of death.

^#^Model 1 was adjusted for age, sex, and education; model 2 was additionally adjusted for APOE ε4 allele and cardiovascular disease in midlife; model 3 included death as a competing risk event with the adjustment of all covariates in model 2.

Abbreviation: CVH, cardiovascular health**.**

**Reviewer #2**

1. In general this is a well-written manuscript. Weaknesses lie in the fact that not all components of the AHA simple 7 score were available, but that a score was still computed and I don't believe that the score in this format has been validated (if it has been, there should be discussion of this). Because of this, I find less value in use of the AHA Simple 7 metric in this paper and think that it would be more valuable to only report the results of individual components.

Answer: We thank the reviewer for the valuable comments, which inspired us to fully address and further clarify this issue. We agree with the reviewer that a limitation of our study is that we were not able to define all the CVH metrics components following the AHA’s initial definitions due to lack of data on diet (actually available only in a small group of the CAIDE cohort, see references no. 38 and 39) and midlife plasma glucose. We tried to address the reviewer’s comments in three ways: (1) We provided head-to-head comparisons of our definitions and those from the original AHA Life’s Simple 7 in Table 1, thus, readers could easily recognize the differences. (2) We have tried to further divide global CVH metric score into behavioural CVH metrics (smoking, physical activity, and BMI) and biological CVH metrics (blood glucose/diabetes, blood pressure, and total cholesterol), as suggested in the AHA’s recommendations and used in the Whitehall II Study (reference no. 16). We believe that this is particularly relevant given that the associations of biological CVH metrics with dementia risk vary with age. (3) We tried to briefly discuss about the potential impact of lack of diet component and midlife plasma glucose in the CVH metrics on the associations of CVH metrics with dementia risk—it generally leads to underestimation of the reduced risk of dementia associated with ideal CVH metrics, according to previous reports from the CAIDE project where dietary data were available on only a subsample of the CAIDE cohort (references no. 38 and 39). Therefore, we have now made major revisions throughout the manuscript in the methods (see page 10), results (see pages 13-16, Tables 3-4, and Fig 2), and discussion sections (see pages 17-18).

2. In general the writing is clear, but there are a few sentences that are not grammatically correct and would benefit from some editing. In general, writing is good.
Answer: We thank the reviewer for kindly pointing out this. Our senior authors have carefully checked the language throughout the manuscript.

3. More detail is needed regarding the definition for physical activity more than twice a week. What was defined as physical activity twice a week? Any physical activity (including walking around the block)? Exercise at a gym? Etc. AHA Simple 7 defines this as >=150 min/week of moderate activity or >=75 min/week of vigorous activity or >=150 min/week of moderate and vigorous activity.
Answer: We thank the reviewer for this valuable comment. In CAIDE, the question on leisure activity is “How often do you participate in leisure‐time physical activity that lasts at least 20–30 minutes and causes breathlessness and sweating?” We categorized the leisure-time physical activity into never (poor), once a week or less (intermediate), twice a week or more (ideal). The definition of an ideal level of physical activity is also in line with our previous study from CAIDE cohort, as we cited now (reference no. 26). We have added this definition to Table 1 and the text in the section of Definition of CVH metrics (page 9).

4. Since the author's score is missing diet and has a proxy measure for fasting plasma glucose, it isn't really the AHA Simple 7 score anymore. Because of this, it may be more prudent to only evaluate individual components of this score and to not include the computed global CVH metrics score, unless this has been validated previously.
Answer: We focused on the composite measurements in this study because individual factors of CVH metrics in mid- and late-life have been extensively investigated in the literature (see references no. 2-4, 8-10 for reviews), whereas few studies have examined the composite CVH metrics in association with risk of dementia (refer to the brief summary of the literature on pages 6-7). However, to fully address the reviewer’s comments, we have now reported the results of individual components of CVH metrics in mid- and late-life in association with risk of dementia in the supplemental tables (see S1 Table and S2 Table). In addition, it is true that the definitions of composite CVH metrics in our study differ from those of the AHA Life’s Simple 7. We have tried to clarify this issue in the ways as we described in details above (please refer to our responses to the comment no. 1 above).

5. In Table 1, it looks like participants in analytical sample 2 (late-life) are survivors (slightly more women, much more ideal physical activity, much more ideal total cholesterol), etc. Can authors discuss this further in their manuscript and what this means for their conclusions? There is some discussion of this, but I think that it could be discussed more extensively.
Answer: We thank the reviewer for the comments. We have now reorganized Table 1 by comparing midlife and late-life characteristics separately between participants included and not included in the analytical sample 2, which was also in response to the comment no. 2 from reviewer #4 (In the revised manuscript, it is now numbered Table 2, as Table 1 reports the definitions of CVH metrics components). We also added brief discussion on the differences of characteristics between the two groups (see page 19).

**Reviewer #3**

1. The study is of relatively small size (at best n=2000 in mid-life) limiting the power of the analysis, and precision of the estimates as shown by the width of the confidence intervals. In particular, this precludes to study with more granularity the patterns of change in CVH between mid-life and late-life: potentially 9 groups of change exist and here only three were investigated. As a corollary, lost to follow-up is substantial: 25% between mid-life and late-life exam1, and an additional lost to follow-up of half the population between late life exam 1 and late life exam 2, so that the analytic sample is likely to be highly selected.
Answer: We agree with the reviewer that the statistical power (sample size) is a potential limitation, as we acknowledged, especially for some subgroup analyses. For the changes or patterns of composite CVH metrics from mid- to late-life, we have now reported the results for all the nine combining groups of CVH metric levels in mid- and late-life in association with dementia risk in Figure 2. Although some subgroups had limited power in this analysis, the figure 2 did show trends of different patterns of CVH metrics in mid- and late-life in association with dementia risk. We acknowledged that these patterns of relationship between CVH metrics in mid- and late-life and risk of dementia deserved further confirmation in the large-scale population-based studies. In addition, The participation rate of 75% (or dropout rate ~25%) in the 1998 examination was generally comparable with that of population-based aging projects in Nordic countries (e.g., participating rate was 76.4% in the Kungsholmen Project and 73.3% in the SNAC-K project in Sweden, reference no. 6). Furthermore, the substantial “dropout rate” from the examination in 1998 to that in 2005-2008 was largely due to death (78.3% of the 517 dropouts were due to death, see figure 1), which can be expected in the prospective cohort studies of older adults. Indeed, the refusal rate was relatively low (~8%). Anyhow, we have acknowledged this as a potential limitation (see page 19).

2. As acknowledged by the authors, diet was missing in their CVH evaluation which limits comparison with other studies based on the full (e.g. 7 items) CVH scale. The authors should discuss to which extent this missing metric impacts their results.
Answer: We thank the reviewer for this comment. We have now added a brief discussion about the potential impact of lack of dietary data on the results on page 19 (also refer to our answer to comment no. 1 from reviewer #2).

3. In that study, there was no significant association between late-life CVH and dementia. The authors concentrate the discussion of this finding by a comparison with a German study that did not find any significant association (ref #19). However, their result is inconsistent with a paper cited in ref #17 that reports significant association with dementia, but the authors did not discuss these inconsistencies.
Answer: We have now added a brief discussion on the inconsistent findings between our study and the literature (reference no. 17), see page 17. We thank the reviewer for kindly pointing out this.

**Reviewer #4**

**Major comments**

1. Methods/Results: In order to further explore components of mid-life and late-life CVH in relation to incident dementia, I suggest the authors consider adding to their investigation models in which all 6 components are added simultaneously as independent variables, rather than the summary CVH score. These models would show the magnitude and direction of the hazard ratio for each component adjusted for the others, and which of the 6 components matter more than others. This could be especially informative for comparing the results pertaining to mid-life CVH (Table 2) and late-life CVH (Table 3), given that body mass index, blood pressure, and cholesterol may behave differently in mid-life versus late-life in relation to dementia risk.

Answer: We thank the reviewer for the valuable suggestions of the additional analyses. In this study we focused on the composite measures of CVH metrics from mid- to late-life, instead of individual components, because there are numerous studies that have investigated those individual factors in midlife and old age in association with late-life risk of dementia. However, we completely agree with the reviewer that results for individual components are also informative for comparison between midlife and older adults. We have now reported the results of individual components of CVH metrics in mid- and late-life in association with dementia in S1 and S2 tables.

In addition, we also agree with the reviewer that certain biological or cardiometabolic factors (e.g., blood pressure and total cholesterol) in midlife and late-life may be differentially associated with late-life risk of dementia. The reviewer’s comment inspired us to consider dividing the composite measure of global CVH metrics into the composite measures of behavioral CVH metrics (smoking, physical activity, and BMI) and biological CVH metrics (blood pressure, total cholesterol, and diabetes/blood glucose), and analyzed separately their associations with dementia risk, which was suggested in the AHA’s recommendations and was analysed previously in the Whitehall II Study (reference no. 16). We have now described this analysis in the methods section (see page 11), added the results to Tables 3-4 and Fig 2, and briefly discussed about these results (see pages 17-18).

2. Results, Table 1: I suggest the authors add two columns to Table 1 to enhance readers' understanding of the participants' characteristics. The first new column would be the mid-life characteristics of the subset of analytical sample #1 who were not included in analytical sample #2 (705 people). The second new column would be the mid-life characteristics of analytical sample #2 (744 people). These two columns could be placed in between the existing columns for analytical sample #1 and analytical sample #2. These columns would help us see whether the two halves of analytical sample #1 were different, and would also help us see more directly the change in CVH over time from mid-life to late-life in analytical sample #2. Also, in conjunction with these additions to Table 1, the second paragraph of the Results section could be deleted entirely, because the information in that paragraph partially matches what I am suggesting to add to Table 1.

Answer: As the reviewer suggested, Table 1 (table 2 in the revision) was now reorganized aiming to compare midlife and late-life characteristics separately between participants included (n=744) and those not included in the analytical sample 2 (n=705). Accordingly, we briefly described the results from the new Table 2 in the section of Results (see pages 12-13). We thank the reviewer for the very valuable suggestions.

3. Results, Table 4: I suggest the authors consider adding a model that includes the mid-life CVH score (per 1-point increment) and the late-life CVH score (per 1-point increment) simultaneously as independent variables, and also possibly the interaction of those two CVH scores. This model would complement the model based on combinations of mid-life and late-life CVH categories.

Answer: We thank the reviewer for the valuable comments. We have now added this analysis on continuous variables (see pages 14-15). We also tested the interactions of the two CVH scores, as continuous variables, on dementia risk, and their interaction was statistically significant (p for interaction = 0.001). Because the interaction of two continuous variables may be difficult to interpret and understand, and is not widely used in medical and public health research (Jaccard J, et al. The Detection and Interpretation of Interaction Effects Between Continuous Variables in Multiple Regression. Multivariate Behav Res. 1990;25(4):467-478), we reported the results of combining midlife and late-life CVH categories (poor, intermediate or ideal) in association with dementia risk (see Fig 2) (also refer to our response to reviewer #1, comment no. 6).

4. Discussion: As the authors note in the paragraph about limitations, this study may suffer from selective survival by levels of CVH. I suggest that the authors also recognize and point out that the study may suffer from selective survival by cognitive decline and dementia. One of the main reasons eligible participants may have not participated in dementia assessment is because they were too cognitively impaired to participate at all, or because they had died as a consequence of dementia, which would be unknown to the authors. I agree with the authors that the magnitude of associations of mid-life CVH and dementia may be under-estimated, and I suggest the authors clarify in their statement that this is likely because of a joint effect of selective survival by CVH level (which can be measured) and selective survival by cognitive decline & dementia (which is unknown for non-survivors and cannot be measured). The first problem, selective survival by CVH level, can be assessed directly in the authors' data, and I suggest the authors conduct this assessment and include the findings in the Results section. For analytical sample #1, compare the mid-life characteristics for those participants who were included in the 1998 dementia assessment versus those who were not. And for analytical sample #2, compare the mid-life and late-life (1998) characteristics for those participants who were included in the 2005-2008 dementia assessment versus those who were not. These analyses will enhance the paper and allow the authors to comment more concretely on the possibility that selective survival influenced the hazard ratios. Fortunately, any bias in the hazard ratios due to selective survival is very likely to be conservative, resulting in under-estimated magnitude of association, rather than anti-conservative.

Answer: We thank the reviewer and completely agree with the reviewer for the very thoughtful comments. Indeed, selective survival related to both CVH metrics and cognitive outcomes might affect the results (usually underestimation of the true associations), as we have now acknowleged (see page 19). For the analytical sample 1, the comparison of midlife characteristics between those included and not included in the 1998 examination were previously reported (reference no 21, i.e. Kivipelto M, et al. BMJ 2001;322:1447-1451). We have now briefly discussed this and cited this previous study. For the analytical sample 2, the comparison of midlife and late-life characteristics between included and excluded people has been added to Table 2, in response to the reviewer’s comment no. 2. Finally, we have reported the cumulative incidence rates of dementia by CVH metric categories. To further address the potential impact of late-life selective survival, we employed the competing risk models (death as a competing risk event) in the analysis. We have reported the results from competing risk models in model 3, Tables 3-4, and Fig 2. Accordingly, we updated the descriptions of methods (page 12), results (pages 13-16), and discussion (page 19) sections.

**Minor comments:**

5. Abstract: The opening statement that associations of CVH with dementia "remain to be explored in cohort studies" seems misleading, given that the authors cited a number of relevant prior publications on CVH and dementia or cognitive impairment or cognitive decline from cohort studies (refs 15-19 cited in the Introduction and refs 28 and 36). I suggest the authors come up with a more appropriate statement for the first sentence of the Abstract.

Answer: We thank the reviewer for this comment. We have rephrased the sentence.

6. Abstract: The authors stated that the cohort study included 1,449 participants followed from mid-life to late-life. I suggest the authors clarify that 1,449 participants were followed from midlife to late-life (1998), and then a subset of 744 were followed further into late-life (2005-2008). Otherwise, readers of the abstract may wonder why there were relatively few cases of incident dementia (47 cases) identified at the 2005-2008 exam, after 61 incident cases were identified at the 1998 exam.

Answer: We have rephrased the description and clarified that in the Abstract, as the reviewer suggeted.

7. Abstract: I suggest the authors consider including hazard ratios of dementia for intermediate CVH in the abstract, in addition to the hazard ratios for ideal CVH.

Answer: We have now completely rewritten the methods and findings section in the abstract due to the major revisions and added the hazard ratio of dementia associated with intermediate CVH metrics to the abstract.

8. Abstract: The Conclusions sentence seems slightly mis-matched to the findings. I think the findings supported lower risk of dementia among people who had good CVH in mid-life (Table 2), those who had good CVH in either mid-life or late-life (Table 4), and those who had good CVH in both mid-life and late-life (Table 4). I do not think the authors reported any finding to support the statement that "improving CVH from mid- to late-life" is associated with lower risk of dementia. I suggest the authors re-work this Conclusions statement to accurately reflect their findings.

Answer: We have completely updated the analyses according to the reviewers’ comments and suggestions. Accordingly, we have now rephrased the conclusions to reflect the key findings from the new analyses. We thank the reviewer for kindly pointing out this.

9. Introduction: After citing refs 15-19, which are cohort studies about cardiovascular health and risk of dementia, the authors assert that "the associations between patterns of CVH metrics from mid- to late-life and risk of dementia remain to be clarified." This sounds vague. What, specifically, remains to be clarified? I suggest the authors revise this sentence to be more specific about the gap in knowledge that remains after refs 15-19, which the present study will address.

Answer: We have modified this sentence (see page 7).

10. Methods, Figure 1: The sample assessed for dementia in 1998 was 1,449 people. The sample in whom late-life CVH was measured in 1998 was 1,426 people. Why are these numbers different? What happened to the 23 people who were assessed for dementia in 1998 but who were not assessed for late-life CVH in 1998? Also, I suggest that the authors note more clearly in Figure 1 that mid-life CVH was assessed in 1972-1987 and that late-life CVH was assessed in 1998.

Answer: In CAIDE, not all participants who were invited for the second wave of late-life examination (2005-2008) were directly from the participants of the first wave of late-life examination in 1998. Actually, the 1426 participants eligible for second late-life examination (2005-2008) were those alive from midlife examination and living in the two areas (Kuopio and Joensuu areas of Eastern Finland). In this study, we selected 744 persons who underwent the 1998 examination, were free of dementia, and completed the 2005-2008 examination (i.e., the analytical sample 2). We have modified both Figure 1 and description of the Study design and participants to make this clear (see pages 7-8).

11. Methods: The authors calculated incidence rates of dementia as number of dementia cases divided by number of person-years of follow-up. This method may be OK, but does not account for the competing risk of death, and therefore the comparison of incidence rates across levels of CVH may be biased due to different rates of death across CVH levels. To overcome this limitation, the authors may want to consider estimating cumulative incidence of dementia from Cox proportional hazards models that account for death as a competing risk. See Andersen PK, et al. Int J Epidemiol. 2012;41:861-870. (In contrast, the hazard ratios for dementia are fine without accounting for the competing risk of death.)

Answer: We thank the reviewer for the valuable comments. As the reviewer suggested, the incidence of dementia was recalculated from Cox regression model after taking into account the competing risk of death. We also analyzed the associations of CVH metrics (in both composite measures in Tables 3 and 4 and individual components in S1 ans S2 Tables) with dementia risk by using the Fine and Gray competing risk regression models taking into account death as a competing risk event. We have described the statistical methods in the part of Statistical analysis (see page 12) and revised the results accordingly (see pages 13-16, Tables 3-4, Fig 2, S1-2 tables).

12. Methods: The authors state that "the proportional-hazards assumption was verified using time-dependent covariates …" If I understand the authors' intent correctly, they added a timeXexposure interaction to the Cox model to allow for the exposure coefficient (hazard ratio) to vary over follow-up time. If so, the proper terminology is "time-dependent coefficient," not "time-dependent covariate." For an explanation of this distinction and of the appropriate method for adding a timeXexposure interaction to the Cox model, see Therneau T, et al. April 2, 2020. cran.r-project.org/web/packages/survival/vignettes/timedep.pdf.

Answer: We thank the reviewer for pointing out this. The reviewer is correct, and we have now revised the sentence (see page 12), as the reviewer suggested.

13. Methods, Table S1: If the journal will allow it, I suggest incorporating Table S1 into the main manuscript instead of putting it in a supplement, as this table is critical for understanding the exposure measures. If the journal limits the main manuscript to a total of 5 tables + figures, then one possibility would be to merge Tables 2 and 3 into a single table to make room for one more table.

Answer: We completely agree with the reviewer and thanks for this suggestion. We have now moved the S1 Table in the submitted version as new Table 1 in the revised manuscript.

14. Results, Table 1: The statistical comparisons of characteristics of analytical sample #1 with characteristics of analytical sample #2, resulting in P values, are probably not strictly valid, because the two samples are not independent, but the methods for calculating the P values (t tests, chi-square tests) probably assume independent samples. If I understand correctly, the participants in analytical sample #2 are a subset of participants in analytical sample #1. The P values are not really meaningful or important, anyway, because the purpose of Table 1 is simply to describe the characteristics of the sample, not to make inference to a population. I suggest the authors omit P values from Table 1 and from the corresponding paragraph in the Results.

Answer: We thank the reviewer for the kind comments and valuable suggestions. We agree with the reviewer and we have now reorganized Table 1 (Table 2 in the revision) (also refer to our response to the reviewer’s major comment no. 2).

15. Results, Table 1: The percentage of participants in the intermediate category of smoking seems quite high, given that this category represents quitting smoking within the past 1 year, which is quite a narrow window given that smokers early in life may quit at any time over the course of decades. How did the authors determine whether former smokers had quit within the past 1 year?

Answer: The category of smoking was in line with the literature (Samieri C, et al. JAMA 2018;320:657-664). Smoking status was defined according to the self-administered questionnaire that included several questions on smoking habits. We determined the quite time of former smokers according to the participant’s response to the question: “When did you smoke your last cigarette?”.

16. Discussion, 3rd paragraph: The authors state that "We found no evidence for the association between late-life CVH metrics and the risk of dementia." While this may be because late-life CVH are less strongly associated with dementia, relative to mid-life (as the authors note), it may also be because of low precision due to small numbers of incident dementia cases in the analysis, relative to the estimated effect sizes (Table 3). The 95% CIs are very wide. I suggest the authors consider whether the precision is adequate in Table 3 for ruling out an effect of late-life CVH, and comment on this in the Discussion.

Answer: We agree with the reviewer that statistical power might be limited to detect a significant association. On the other hand, it has been well established that there is an age-dependent relationship of several CVH metrics components (e.g., high blood pressure, high total cholesterol, and high BMI) with dementia risk, such that these CVH components, if occurring in midlife, but not necessarily in later in life (e.g., >65 years), are associated with late-life dementia. The potential biological explanation for the lack of association between late-life CVH risk factors and risk of dementia is partially due to reverse causality (references no. 31-33). We have now briefly discussed this (see pages 17-18).
