# Supplementary material for: Cardiovascular health metrics from mid- to late-life and risk of dementia: A population-based cohort study in Finland
Source: PLoS Med. 2020 Dec 15;17(12):e1003474. doi: 10.1371/journal.pmed.1003474 (PMC7737898; doi:10.1371/journal.pmed.1003474)
Supplement: Attachment — Submitted filename: Final_Response to reviews_2020Oct5.doc [file pmed.1003474.s007.doc]

**Editors’ comments**

1. Thank you for agreeing to make your data available. However, PLOS policy does not allow authors to be the primary contact for data access. Please provide a different contact for researchers applying for data access, e.g. a member of the CAIDE data management and maintenance committee who is not a study author.

Answer: We have changed the contact person in data availability statement.

2. Thank you for providing your pre-specified analysis plan and noting the changes or additions made. In the “Difference in analyses between planned and performed” section, please also note the reasons for the change (e.g. unexpected pattern in the data or in response to a reviewer request).

Answer: We have added the reason for the change of analyses between the planned and performed analyses, see S1 study protocol.

3. Title: please include the country “Cardiovascular health metrics from mid- to late-life and risk of dementia: a population based study *in Finland*”

Answer: We have added the country to the title, as suggested.

4. Abstract Methods and Findings, second sentence: is it more accurate to say “We defined and scored *six of the seven* components of global CVH metrics…”?

Answer: We have modified the sentence following the suggestion, see page 3.

5. Please provide p-values alongside 95% CIs throughout the manuscript.

Answer: We thank the editor for the suggestions. We have now added the p values alongside the 95% CI in the text. In addition, we added notes under the tables to indicate the p values for 95% CI (i.e., p<0.05 or p<0.01), see tables 3-4, S1 table.

6. Abstract Conclusions: Please address the study implications without overreaching what can be concluded from the data; the phrase "In this study, we observed ..." may be useful. Similarly for the first sentence of the Discussion Conclusions.

Answer: We have modified the sentences as suggested. See pages 4 and 20. We thank the editor for the kind suggestion.

7. Was informed consent written or verbal? Please specify when mentioned in the Methods.

Answer: We have clarified that the consent was the verbal informed consent for midlife surveys in 1972-1987 and the written informed consent for late-life surveys in 1998 and 2005-2008, see page 8.

**Comments from the reviewers:**

**Reviewer #1**:

We thank the authors for addressing the points raised in the previous review round, in particular the concern relating to competing risks. The additional Fine-Gray model results appear to generally affirm the previously-observed correlations regarding dementia outcomes between poor & intermediate/ideal CVH, for the main global CVH analysis.

However, the relatively low numbers of dementia cases, especially after stratification into subgroups as presented in Figure 2, has resulted in wide hazard ratio confidence intervals that leave some room for doubt, as well as seemingly counterintuitive findings such as intermediate/ideal biological CVH reported to have a HR of >7 compared to poor biological CVH. While possible explanations about biological CVH components such as blood pressure and total cholesterol have been suggested in the discussion, it would seem that the more fine-grained findings would indeed be well-served through larger-scale studies.

Nonetheless, the manuscript appears improved especially having taken into account suggestions from all reviewers, though there remain some minor issues:

1. Further details about the Fine-Gray competing risk model might be provided, possibly in supplementary material. In particular, how was the risk of death incorporated/quantified in the model?

Answer: We thank the reviewer for the kind suggestion. We have now provided a more detailed description on the methods of Fine-Gray competing risk regression models in the supplementary material, see S1 statistical methods.

2. The added statement of APOE being a genetic factor for dementia and being involved in lipid and lipoprotein metabolism (Page 12) might be supported by suitable references.

Answer: We have now added two references supporting this statement, see references 29 and 30.

3. From S1 table, it appears that intermediate smoking (vs. poor) is also significantly associated with reduced risk of dementia (Page 14).

Answer: We have now emphasized this in the results section, see page 14.

4. "having quitted more than one year" might be "having quitted for more than one year" (Page 9), and "Compared with people having ~~the~~ poor global CVH metrics in midlife" might be "Compared with people having poor global CVH metrics in midlife" (Page 13)

Answer: We have modified the sentences as the reviewer suggested, see pages 9 and 13. We thank the reviewer for the kind suggestions of editorial revisions.

**Reviewer #2:**

I appreciate the authors' thorough and responsive revision, including the authors' separation of global CVH metrics into behavioral and biological metrics and the addition of the S1 and S2 tables. I have only a few remaining minor comments:

1) In the introduction, the AHA's simple 7 are introduced, and, separately, the authors introduce that they will be evaluating CVH metrics. At the point of the introduction, it would be helpful to introduce that this paper will be evaluating the Life's Simple 7 factors, categorized as behavioral (sans diet - important to mention here) and as biological. With the recent revisions, the introduction of the Life's Simple 7 here doesn't quite tie into the rest of the paper because it isn't clear at the stage of the intro whether authors are going to evaluate the Life's Simple 7 verbatim or some other CVH metrics. Fine to refer to the Life's Simple 7 but make it clear that this is the background for the work, and what is being done in this paper, and how it's similar/different. This definition comes in the "Definition of CVH metrics" section later in more detail, but at the stage of the intro, I recommend you add a simple short sentence summarizing this exposure and how it's different/similar to Simple 7.

Answer: We thank the reviewer for the valuable comments. We have now added brief information in the introduction emphasizing the knowledge gap of differentiating behavioral and biological cardiovascular metrics, see page 7.

**Reviewer #3:**

The novelty of the study lies in the investigation of pattern of change in cardiovascular health from midlife to late life with incident dementia. However and despite the answers of the authors, to my view, the paper still suffers from the following major limitations:

- the analysis relies on 6 instead of 7 items of the cardiovascular health; the definition of the diabetic metric is incomplete

Answer: We understand the reviewer’s concern. We have now clearly stated in both the abstract (see page 3) and the methods (see page 9) that the composite global CVH metrics were based on six of the seven components included in the AHA’s original recommendations. The reviewers’ comment also inspired us to further separate the global CVH metrics into behavioral and biological CVH metrics (this analysis was not included in our initial analysis plan, as we specified in S1 study protocol). In this regard, we thank the reviewer for the valuable comments, which helps make our study novel and more informative.

We agree with the reviewer that the lack of diet data as well as the proximal definition of blood glucose categories in midlife is a limitation. We have acknowledged this limitation and briefly discussed the potential impact on the results, see page 19.

- there is too much attrition over time: 50% of the eligible population is lost from 1998 to 2005.

Answer: Indeed, out of the 1449 participants in 1998, 705 were excluded, leaving 744 in the analytical sample 2 (see Fig 1 flowchart of study participant for details). Out of the 705 excluded, 405 (57.4%) were due to death, which may largely reflect the nature phenomenon of the follow-up study of an aged cohort, and additional 61 persons (8.6%) were excluded due to dementia determined at the 1998 examination because we considered this wave of examination as baseline when we examined the longitudinal associations of late-life CVH metrics or patterns in CVH metrics from mid- to late-life with subsequent risk of dementia (i.e., a cohort of free from dementia at baseline). One-third of the attrition (33.9%) was due to refusal (n=135, 19.1%) or incomplete data for the diagnosis of dementia (n=104, 14.8%) in the 2005-2008 examination. Nevertheless, we compared the midlife and late-life characteristics of included and excluded participants in the analytical sample (see page 13, Table 2).

- attrition can be handled with IPW technics but this was not done

Answer: We thank the reviewer for the comment. We did not apply the IPW techniques to the data analysis because nearly two-thirds (66%) of the attrition was due to death (57.4%) or diagnosis of dementia in 1998 (8.6%) (see our response above), and we have calculated the cumulative incidence and used the competing risk regression models to account for the competing risk due to death.

Indeed, the IPW techniques have been used to account for the attrition-related bias in some observational studies or controlled trials (e.g., Sommerlad A, Sabia A, et al. PLOS Med 2019). Given that we have controlled for a range of potential confounders, and additionally, the major part of attrition due to death has been taken into consideration in our analyses, we feel that the potential confounding bias and attrition related bias have been properly controlled for. In addition, it has been argued that from the public health perspective, the results from the real population (i.e., unweighted) may be interesting (Chaix B, Evans D, Merlo J, Suzuki E. Weighing up the dead and missing: reflections on inverse-probability weighting and principal stratification to address truncation by death. Epidemiology. 2012;23:129-31; discussion 132-7). However, we are open to provide results from IPW weighed analysis if the reviewer prefers us to do it.

- the change analysis (which is the novel aspect of the paper) is based on 47 incident dementia cases only

Answer: We agree that overall, the statistical power is limited for the analysis of changes of CVH metrics from mid- to late-life (in total 9 groups) and subsequent risk of dementia. However, we have tried to make our conclusions on groups that had proper power showing statistically significant results (e.g., intermediate global CVH metrics in both midlife and late-life, intermediate behavioral CVH metrics in both midlife and late-life, ideal behavioral CVH metrics in both midlife and late-life), and we were very cautious to make any conclusions for the groups that did not have sufficient power (e.g., midlife ideal and late-life poor global/behavioral/biological CVH metrics, and midlife poor and late-life ideal behavioral/biological CVH metrics). While we have acknowledged the limitation of having insufficient power for certain subgroup analyses (e.g., even no dementia case), we also emphasized the need of future large-scale studies to verify the key findings from our study, see page 20.

- the results are hard to follow and to interpret, especially those on the change in cardiovascular health.

Answer: We have reorganized Fig 2 as a two-dimensional figure (in response to the comments from reviewer 4 below). In addition, we have carefully discussed the main findings with regard to the relations of CVH metric patterns from mid- to late-life with dementia risk (see page 18). We hope that this may help to easily follow the results.

**Reviewer #4:**

In my opinion, the authors revisions in response to peer review comments—mine and other reviewers—resulted in an improved manuscript. I have no major comments and a few minor comments:

MINOR COMMENTS:

1. Table 2 (formerly Table 1) is now more informative then before about the nature of the sample. I still think the hypothesis test and P values are not very meaningful, given that the purpose of this table is simply to describe the characteristics of the sample, not to make inference to the population. The authors could consider simplifying Table 2 by omitting the P value columns.

Answer: We agree that one of the aims of table 2 is to show mid- and late-life characteristics of the sample. In addition, table 2 aims to compare the differences in the mid- and late-life characteristics between those excluded and included in the analytical sample 2. In this regard, the p values might be relevant. However, we are open to remove the p value columns in table 2 if the reviewer or editor prefers.

2. The addition of cumulative incidence estimates accounting for competing risk of death to Tables 3 and 4 is informative. I suggest clarifying a few details about these estimates: (a) The length of follow-up for cumulative incidence should be reported, such as "20-year cumulative incidence," or whatever number of years of follow-up the estimates correspond to. (b) The word "rate" is not needed for cumulative incidence and could be omitted from the table column heading; the measure is "X-year cumulative incidence (%)," indicating the total incidence over the time span, not a rate. (c) Whether the cumulative incidence was adjusted for Model 1 or Model 2 covariates should be specified.

Answer: We thank the reviewer for the precision suggestions for revision. As the reviewed suggested, we added the year before cumulative incidence and deleted the word “rate”. We also specified under the table that the cumulative incidence was calculated from the crude Cox models, see tables 3-4.

3. Figure 2 is interesting, but highlights clearly the lack of precision for estimating hazard ratios across so many different combinations of midlife and late life cardiovascular health levels (due to very small sample size for most groups). I know I suggested the authors undertake a more thorough analysis of the combination of midlife and late life cardiovascular health levels, and I am grateful they did so, but now I can see that the sample size is inadequate for such analysis to be very meaningful. The confidence intervals for most bars in Figure 2 are so wide that it is difficult to draw conclusions. Showing the confidence intervals visually instead of with numbers would enhance the figure, but would be difficult to show in the 3-dimensional layout. Thus, I suggest the authors consider revising Figure 2 into a 2-dimensional layout and show the height of the confidence intervals visually on the vertical axis rather than as printed numbers over the bars. Finally, the resolution of Figure 2 was very poor, such that the figure was nearly impossible to read. The authors should provide a high-resolution figure for publication.

Answer: We again thank the reviewer for the previous and current suggestions, which inspired us to thoroughly analyze our data. For the limitation of statistical power for some subgroup analyses, please refer to our responses to reviewer #3 above. Following the reviewer’s suggestion, we have modified the Fig 2 into two-dimensional layout and showed the confidence intervals as well, see Fig 2. We have also reformatted the Fig 2 to increase the resolution of the figure.

4. Tables S1 and S2 are informative because they shed light on the heterogeneous influence of the 6 factors on risk for dementia. Table S2, in particular, helps us see one potential explanation for the late-life cardiovascular health score not being associated with lower dementia risk—"ideal" levels of late-life blood pressure, blood cholesterol, and body mass index are all associated with higher dementia risk, not lower risk. This is in line with prior evidence and especially helps to explain the findings illustrated in the bottom panel of Figure 2 (biological subscore). The authors addressed this briefly in the first paragraph of the Discussion; they could consider expanding this discussion a bit to highlight specific factors that behave differently in late life than in midlife for predicting dementia risk, and implications for using/interpreting the AHA cardiovascular health summary score for late-life data.

Answer: We thank the reviewer for the suggestion. We have indeed mentioned that specific biological CVH metric components (e.g., blood pressure and total cholesterol) in middle age and late-life were differentially associated with late-life risk of dementia, see the 4th paragraph of discussion (pages 17-18). As the reviewer suggested, we have now emphasized that the age-varying associations of individual biological CVH metrics with dementia risk should be kept in mind when interpreting results of the late-life composite CVH metric score in relation to dementia risk, see page 18.

5. I agree with the authors' decision to omit the analysis of population attributable risk, for the reasons they gave in their response to Reviewer #1 comment (5).

Answer: We thank the reviewer for this comment.
